# Rectus muscle diastasis in Italian women: determinants of disease severity, and associated disorders

**DOI:** 10.3389/fsurg.2024.1360207

**Published:** 2024-03-11

**Authors:** Alberto Sartori, Ahmad Tfaily, Emanuele Botteri, Jacopo Andreuccetti, Enrico Lauro, Gulser Caliskan, Giuseppe Verlato, Alberto Di Leo

**Affiliations:** ^1^ U.O. Chirurgia Generale e d’Urgenza, Ospedale Montebelluna, Montebelluna, Italy; ^2^ Unit of Epidemiology and Medical Statistics, Department of Diagnostics & Public Health, University of Verona, Verona, Italy; ^3^ General Surgery, ASST Spedali Civili di Brescia, Montichiari, Italy; ^4^ UOC Chirurgia Generale 2, ASST Spedali Civili di Brescia, Brescia, Italy; ^5^ U.O. Chirurgia Generale, Ospedale Civile Santa Maria del Carmine, Rovereto, Italy; ^6^ U.O. Chirurgia Generale e Mininvasiva, Ospedale San Camillo, Trento, Italy

**Keywords:** diastasis recti abdominis, risk factor, incontinence, prolapse, hernia abdominalis

## Abstract

**Purpose:**

Diastasis of rectus abdominis (DRA) refers to a separation of the rectus abdominis from the linea alba, which is common in the female population during pregnancy and in the postpartum period. The present study aimed at investigating DRA severity, risk factors and associated disorders.

**Methods:**

In the present cross-sectional study, a web-based questionnaire was addressed to the 23,000 members of the Women's Diastasis Association. The questionnaire comprised three parts, dedicated respectively to diastasis characteristics, possible risk factors, and related disorders. Faecal and urinary incontinences were assessed using the Wexner and ICIQ-SF score, respectively. Risk factors for diastasis severity (<3, 3–5, >5 cm) were evaluated by a multinomial regression model.

**Results:**

Four thousand six hundred twenty-nine women with a mean age (SD) of 39.8 (6.5) years and a median BMI of 23.7 kg/m^2^ (range 16.0–40.0) responded to the questionnaire. Proportion of DRA >5 cm increased from 22.8% in norm weight women to 44.0% in severely obese women, and from 10.0% in nulliparous women to 39.3% in women with >3 pregnancies. These associations were confirmed in multivariable analysis. DRA severity was associated with the risk of abdominal hernia and pelvic prolapse, whose prevalence more than doubled from women with DRA <3 cm (31.6% and 9.7%, respectively) to women with DRA >5 cm (68.2% and 20.2%). In addition, most patients reported postural pain and urinary incontinence, whose frequency increased with DRA severity.

**Conclusion:**

The present study confirmed that DRA severity increases with increasing BMI and number of pregnancies. Larger separation between rectal muscles was associated with increased risk of pain/discomfort, urinary incontinence, abdominal hernia and pelvic prolapse. Prospective studies are needed to better evaluate risk factors.

## Introduction

Diastasis of rectus abdominis (DRA) refers to a separation of the rectus abdominis from the linea alba, which often affects multipara women (1). There is no universal definition of the minimum distance to diagnose DRA, although two papers in the literature agree on a minimum inter-recti distance of 20 mm supraumbilical regardless of patient age (2–4).

Although the whole population can be affected, DRA has a marked prevalence in women, especially after multiple births. Indeed, pregnancy changes the abdominal conformation due to the space occupied by the uterus and increases the physiological lumbar lordosis. This changes the levels of elongation of the abdominal muscles by modifying the angles of origin and insertion, leading, among other effects, to flaccidity of the linea alba with a consequent increase in the space between the muscle's bellies of the rectus (5–7). This effect may persist from 24% to 70% of cases during postpartum in different regions of the linea alba. The remodelling time typically lasts 6–8 weeks, although this parameter may vary individually with continuous decreases up to 6–12 months postpartum (6, 8, 9). The prevalence of this pathological condition of the abdominal wall is very variable, ranging from 39% to 52% in post-pregnancy women. In addition to pregnancy, obesity is a major risk factor for the onset of this condition (10).

An increase in the distance between the anterior borders of the rectus muscles influences the strength of the abdominal wall musculature and does not usually cause pain at rest. During physical activities, however, the characteristic bulging of the abdominal wall may appear, due to an increase of the intra-abdominal pressure. Therefore, DRA may be associated with epigastric and umbilical hernias. Pregnancy may cause herniation or render a pre-existing one apparent, because of progressively raised intra-abdominal pressure (11–13). The presence of diastasis of the rectus muscles is not only an “aesthetic” fact but is associated with the development over time, if not corrected properly, of uterine prolapse, faecal and urinary incontinence. Considering the role of abdominal muscles in maintaining posture and their engagement in various physical activities one may suspect that the presence of DRA may have an impact on the trunk and pelvic stabilization, thus leading to poor posture, limitations during physical activity, as well as lumbo-pelvic pain and hip pain (13).

There is a scant knowledge on the prevalence, risk factors, prevention, or management of the abovementioned condition. For many years, abdominal diastasis of the rectus was considered an exclusively aesthetic problem by surgeons, and treatment was entrusted exclusively to plastic surgeons. Over the last few years, surgeons' attention to this anatomical, pathological condition has progressively increased. The association of DRA with the presence of a midline hernia has led surgeons to develop some minimally invasive surgical techniques to treat this pathology.

The aims of the present study are:

(1) To investigate possible determinants of the severity of DRA, including maternal conditions, comorbidities/treatments, previous pregnancies, childbirth characteristics.
(2) To study the association between DRA severity and post-partum disorders, such as pain/discomfort, urinary/faecal incontinence.
(3) To address the association between rectal muscle separation and other consequences of increased intra-abdominal pressure, such as hernia, prolapse.

## Materials and methods

In the present observational cross-sectional study, approximately 23,000 Italian women, belonging to the voluntary organisation Diastasi Donna® ODV (https://www.diastasidonna.it/diastasi-donna-odv/), were invited to anonymously answer an online questionnaire by Google form. The questionnaire was filled in by 4,629 women (about 20.1%) aged 39.8 ± 6.5 years (mean ± SD; range 22–74) with a median BMI of 23.7 kg⁄m^2^ (range 16.0–40.0), who had had a median of 2 pregnancies (range 0–5). While filling-in the anonymous questionnaire, women were also asked to release informed consent for anonymous data publication.

The questionnaire comprised three parts, dedicated respectively to diastasis characteristics, possible risk factors, and related disorders (Table 1; Supplementary Table S1). Faecal incontinence was assessed by Wexner score, and urinary incontinence by ICIQ-SF score (14, 15).

**Table 1:** Determinants of severity of rectus abdominis separation.

	Separation of rectos abdominis			*P*-value*
	<3 cm	3–5 cm	>5 cm	
	(*n* = 813)	(*n* = 2,539)	(*n* = 1,277)	
Age at interview, years	39.0 ± 6.8	39.5 ± 6.3	**41.1 ± 6.7**	<0.001
BMI^a^				**<0** **.** **001**
Underweight	39 (**4.8%**)	86 (3.4%)	29 (2.3%)	
Normoweight	548 (**67.4%**)	1,550 (**61.1%**)	619 (48.5%)	
Overweight	170 (20.9%)	661 (26.0%)	410 (**32.1%**)	
Obese	48 (5.9%)	185 (7.3%)	168 (**13.2%**)	
Severely obese	8 (0.9%)	57 (2.2%)	51 (**4.0%**)	
Term pregnancies				**<0.001**
0	9 (**1.1%**)	9 (0.4%)	2 (0.2%)	
1	300 (**36.9%**)	614 (24.2%)	170 (13.3%)	
2	406 (49.9%)	1,511 (**59.5%**)	776 (**60.8%**)	
3	80 (9.8%)	335 (13.2%)	262 (**20.5%**)	
≥4	18 (2.2%)	70 (2.8%)	67 (**5.3%**)	
Caesarean sections/vacuum-assisted deliveries				**<0.001**
0	396 (**49.6%**)	1,045 (**43.3%**)	433 (35.3%)	
1	220 (**27.6%**)	626 (**25.9%**)	228 (18.6%)	
2	154 (19.3%)	631 (26.1%)	439 (**35.8%**)	
≥3	28 (3.5%)	112 (4.6%)	127 (**10.3%**)	
Smoking habits				0.179
Never smoker	413 (50.9%)	1,280 (50.4%)	599 (46.9%)	
Ex-smoker	245 (30.2%)	789 (31.1%)	406 (31.8%)	
Current smoker	154 (19.0%)	469 (18.5%)	271 (21.2%)	
Diabetes^b^	13 (1.6%)	35 (1.4%)	36 (**2.8%**)	0.032
Gestational diabetes^b^	102 (12.6%)	334 (13.2%)	208 (**16.3%**)	0.012
Collagen disease	5 (0.6%)	17 (0.7%)	6 (0.5%)	0.526
Thyroid disease				
Hypothyroidism	87 (10.7%)	321 (12.6%)	180 (14.1%)	0.075
Hyperthyroidism	25 (3.1%)	83 (3.3%)	54 (4.2%)	0.241
Steroids before gestation	37 (4.6%)	92 (3.6%)	51 (4.0%)	0.480
Steroids during gestation	56 (6.9%)	177 (7.0%)	106 (8.3%)	0.266
Kristeller's manoeuvre	142 (19.8%)	528 (**23.5%**)	287 (**25.4%**)	**0.021**
Episiotomy/laceration	314 (40.5%)	1,018 (41.7%)	464 (37.8%)	0.074

Results are reported as mean ± SD for the continuous variable (age), and as absolute frequency (column percent frequency) for qualitative variables.

^a^ Underweight, normoweight, overweight, obese, severely obese: BMI <18.5, 18.5–24.9, 25–29.9, 30–34.9, ≥35 kg/m^2^.

^b^ Information on smoking habits, diabetes, gestational diabetes, collagen disease, Kristeller's manoeuvre and episiotomy was missing in 3, 104, 40, 1,073, 538, 182 women, respectively.

* Significance of differences was evaluated by Kruskal–Wallis test for quantitative variables (actual age, BMI, number of term pregnancies, number of caesarian sections) and by Fisher's exact test for qualitative variables. Significant values are highlighted in bold.

Rectal muscle separation was measured during Valsalva maneuver by ultrasounds and/or computed tomography. In detail, 3,742 women (80.8%), reported to have undergone ultrasounds, 434 (9.4%) abdominal magnetic resonance imaging, and 453 (9.8%) abdominal CT-scan. According to the classifications proposed by Ranney, the severity of DRA was defined by the distance between rectal muscles (mild DRA >3, moderate DRA 3–5, severe DRA >5 cm) and the extension of DRA (supra-umbilical, sub-umbilical, both) (16). Of note, severe diastasis >5 cm is considered as an indication for surgical treatment (13).

### Statistical analyses

The endpoint of the study was separation of rectus abdominis muscles. Significance of the association between DRA severity and potential determinants was evaluated by Fisher's exact test or chi-squared test for categorical variables, and Kruskal–Wallis test for quantitative variables.

In multivariable analysis determinants of DRA severity (≥3, 3–5, >5 cm) were investigated by a multinomial logistic regression model, where BMI, number of pregnancies, Kristeller's manoeuvre and episiotomy were the potential determinants, and age at interview, smoking habits, gestational diabetes, hypothyroidism, use of steroids during gestation the possible confounders.

The association between DRA severity and related disorders (postural pain/discomfort, urinary or faecal incontinence, abdominal hernia, pelvic prolapse) was investigated by Fisher's exact test or chi-squared test. These associations were further investigated by multivariable logistic models, where abdominal hernia (0 = no, 1 = yes), pelvic prolapse (0 = no, 1 = yes) or urinary incontinence (0 = no, 1 = yes) was the response variable, DRA severity was the main determinant, and BMI, number of pregnancies, Kristeller's manoeuvre, episiotomy, age at interview, smoking habits, gestational diabetes, hypothyroidism, use of steroids during gestation were potential confounders.

## Results

### Determinants of severity of rectus abdominis diastasis

In almost two-thirds of the participants (2,983/4,629 = 64.4%) diastasis affected both the supra- and sub-umbilical areas. Diastasis width was moderate (3–5 cm) in 54.9% of cases, and severe (>5 cm) in 27.6%.

The separation between rectal muscles increased with age at interview, BMI, and number of term pregnancies (*p* < 0.001). Separation >5 cm was recorded in 18.8%, 22.8%, 33.0%, 41.9%, 44.0% of underweight, norm weight, overweight, obese, severely obese women, respectively (Table 1). As regards number of term pregnancies, separation >5 cm was observed in 10.0% of nulliparous women, 27.1% of women with 1–3 term pregnancies and 43.2% of women with 4 or more term pregnancies. This trend became even steeper when considering caesarean sections or otherwise assisted deliveries, as the proportion of separation >5 cm increased from 23.1% in women never undergoing caesarean delivery to 47.6% in women undergoing 3 or more caesarean deliveries. The proportion of diabetes increased from 1.6% in women with separation <3 cm to 2.8% in women with separation >5 cm (*p* = 0.032); likewise, the proportion of gestational diabetes increased from 12.6% to 16.3% (*p* = 0.012). Among delivery characteristics, Kristeller's manoeuvre was associated with larger separation between Rectus Abdominis: the manoeuvre was reported by 19.8% of women with separation <3 cm and by 25.4% of those with separation >5 cm (*p* = 0.021). On the contrary, a negative trend was observed between diastasis severity and episiotomy/laceration from delivery, although the association did not achieve statistical significance (*p* = 0.074). Severity of rectal muscle separation was not significantly affected by smoking habits, collagen or thyroid disease, steroid treatment whether administered before or during pregnancy (Table 1).

**Figure 1:**
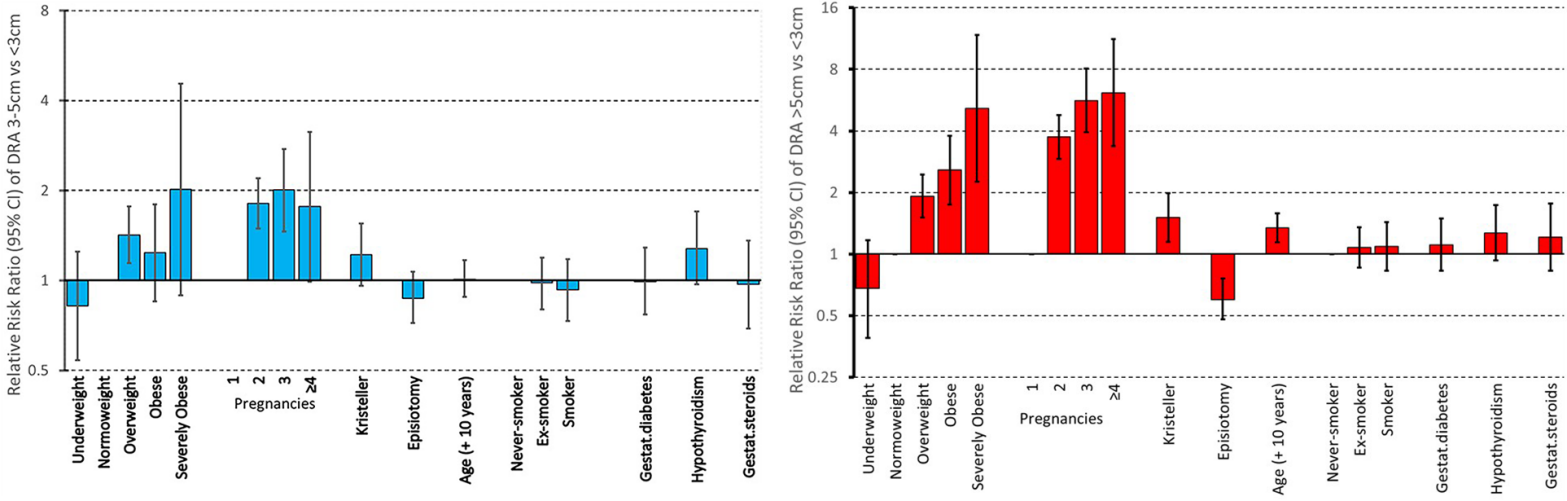
Multivariable analysis of determinants of severity of rectal muscle separation. Relative Risk Ratios (RRR) and *p*-values were computed by a multinomial model, controlling for all other variables. Nulliparous women (*n* = 20) were excluded from the analysis.

The risk profile was substantially confirmed by multivariable analysis: in the multinomial logistic regression model, when considering separation >5 cm vs. <3 cm (reference), the relative risk ratios (RRRs) for overweight, obese, severely obese with respect to normoweight were respectively 1.92 (95% CI 1.51–2.45), 2.57 (95% CI 1.74–3.79), 5.15 (95% CI 2.26–11.77). Rectal muscle separation increased with increasing number of pregnancies: with respect to one pregnancy, the RRRs of 2, 3, ≥4 pregnancies were 3.74 (95% CI 2.91–4.79), 5.64 (95% CI 3.94–8.06), 6.15 (95% CI 3.37–11.23) respectively (Figure 1, lower panel). Kristeller's manoeuvre, which increases intra-abdominal pressure during delivery, was associated with larger separation (RRR of presence vs. absence of previous Kristeller's manoeuvre = 1.51, 95% CI 1.15–1.98), while an inverse relation was observed for episiotomy, which facilitates delivery (RRR of presence vs. absence of previous episiotomy = 0.60, 95% CI 0.48–0.76) (Figure 1, lower panel). Rectal muscle separation significantly increased with increasing age at interview: when considering separation >5 cm vs. <3 cm, the RRR for 10-year increase in age was 1.34 (1.14–1.58). Severity of rectal muscle separation was not significantly affected by either smoking habits, gestational diabetes, hypothyroidism, or corticosteroids treatment during pregnancy (Figure 1).

### Association between severity of rectus muscle diastasis and post-partum disorders: urinary and faecal incontinence, postural pain/dicomfort

The prevalence of post-delivery disorders significantly increased with increasing separation of rectal muscles. The prevalence of pain/discomfort, urinary incontinence, incontinence for gas, incontinence for liquid stools was 50.9%, 64.8%, 70.5%, 33.8% respectively in the group with separation <3 cm and rose to 80.7%, 75.2%, 77.1% and 37.9% in the group with separation >5 cm. At variance, incontinence for solid stools was not significantly affected by DRA severity (Table 2).

**Table 2:** Association between severity of rectus abdominis diastasis and post-partum disorders.

	Separation of rectos abdominis			*P*-value
	<3 cm	3–5 cm	>5 cm	
Pain/discomfort	414 (**50.9%**)	1,782 (**70.2%**)	1,031 (**80.7%**)	**<0** **.** **001**
Urinary incontinence	527 (**64.8%**)	1,771 (**69.8%**)	960 (**75.2%**)	**<0** **.** **001**
Fecal incontinence				
Gas	573 (**70.5%**)	1,900 (**74.8%**)	984 (**77.1%**)	**0** **.** **003**
Liquid	275 (**33.8%**)	861 (**33.9%**)	484 (**37.9%**)	**0** **.** **038**
Solid	257 (31.6%)	789 (31.1%)	422 (33.1%)	0.466

*P*-value was computed by chi-square for trend.

Significant values are highlighted in bold.

The effect of severe DRA on urinary incontinence persisted in multivariable analysis, although the association was stronger in univariable analysis (OR of severe vs. mild diastasis = 1.64, 95% CI 1.36–1.99) than in multivariable analysis (OR = 1.30, 1.04–1.62). Indeed, obesity and episiotomy were much stronger determinants of urinary incontinence than DRA severity. The risk of urinary incontinence was also increased in overweight, older women with several term pregnancies, current and ex-smokers, and women with hypothyroidism (Table 3).

**Table 3:** Multivariable analysis of determinants of urinary incontinence.

	Urinary incontinence	
	OR (95% CI)	*P*-value
Rectal muscle separation		
<3 cm	1 (reference)	
3–5 cm	1.15 (0.95–1.39)	0.150
>5 cm	**1.30** **(1.04–1.62)**	**0** **.** **021**
BMI		
Underweight	0.91 (0.63–1.31)	0.612
Normoweight	1 (reference)	
Overweight	**1.55** **(1.31–1.85)**	**<0** **.** **001**
Obese	**2.11** **(1.56–2.86)**	**<0** **.** **001**
Severely obese	**3.61** **(1.93–6.75)**	**<0** **.** **001**
Kristeller: Yes vs. No	1.17 (0.95–1.43)	0.132
Episiotomy: Yes vs. No	**2.09** **(1.76–2.48)**	**<0** **.** **001**
Age (per 10-year increase)	**1.42** **(1.26–1.60)**	**<0** **.** **001**
Smoking habits		
Never-smoker	1 (reference)	
Ex-smoker	**1.22** **(1.04–1.43)**	**0** **.** **016**
Current smoker	**1.57** **(1.29–1.92)**	**<0** **.** **001**
Gestat. diabetes: Yes vs. No	1.08 (0.88–1.33)	0.474
Hypothyroidism: Yes vs. No	**1.31** **(1.05–1.64)**	**0** **.** **018**
Steroids in pregnancy	1.07 (0.81–1.40)	0.630

Odds ratios (OR) and *p*-values were computed by a logistic model, controlling for all other variables.

Significant values are highlighted in bold.

Nulliparous women were excluded from multivariable analysis.

**Figure 2:**
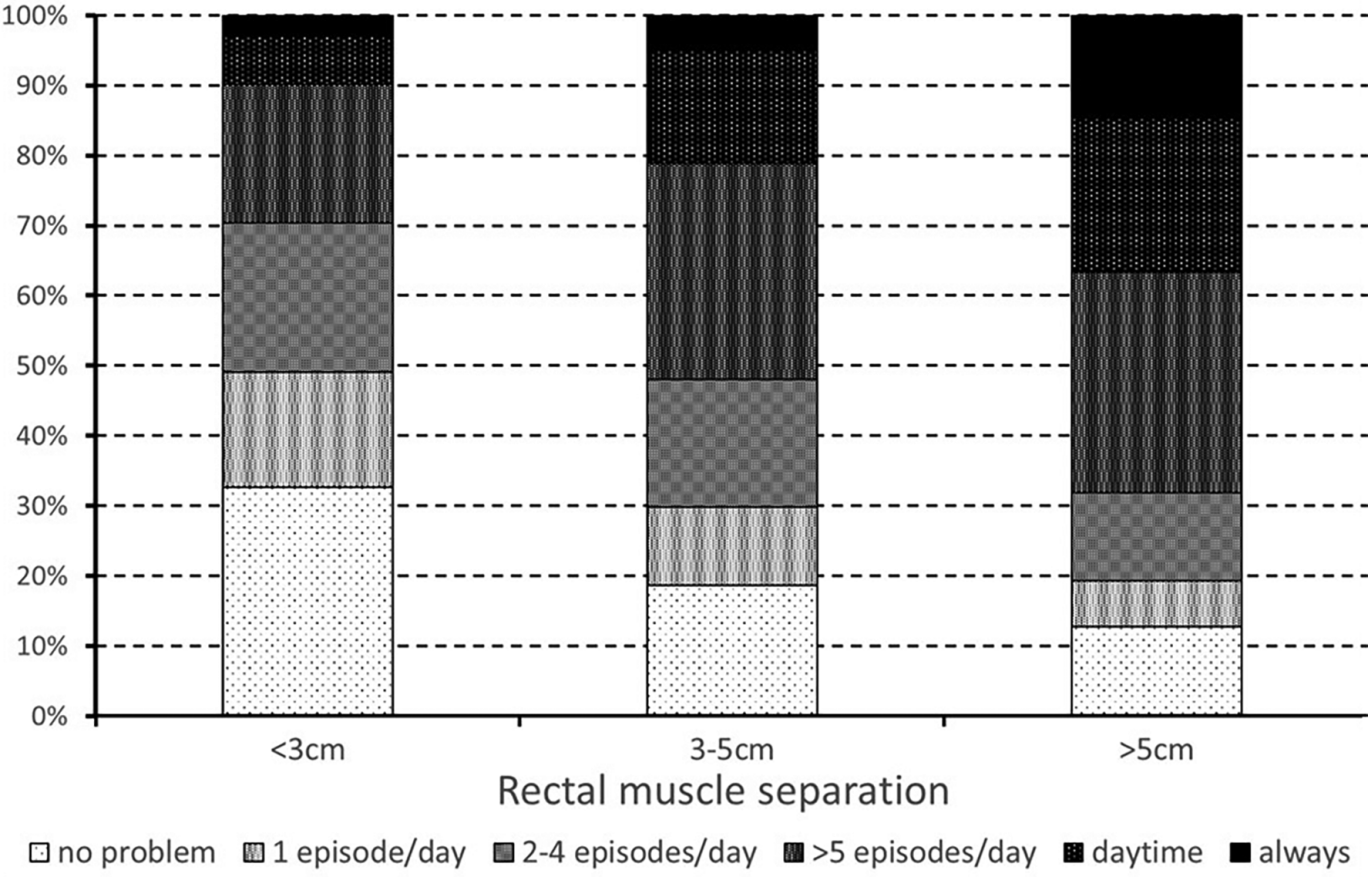
Frequency of postural disorders as a function of rectal muscle separation.

Severity of DRA was strongly associated with the frequency of postural disorders (Spearman's rho = 0.273; *p* < 0.001). Indeed, nearly one third (32.7%) of women with mild DRA <3 cm reported no postural problems and <10% had symptoms always or during the whole daytime. These percentages were reversed in women with severe DRA >5 cm, as only 12.8% reported no postural problems, and 36.7% had symptoms always or during the whole daytime (Figure 2).

### Association between severity of rectus abdominis diastasis and abdominal hernia/pelvic prolapse

Hernia and prolapse were rather frequent disorders in the population under study, affecting 54.4% and 14.6% of women, respectively. Both were strongly correlated with the severity of rectus abdominis diastasis (*p* < 0.001). Indeed, the prevalence of hernia increased from 31.6% in women with separation <3 cm, to 54.8% in women with 3–5 cm separation, and further to 68.2% in women with separation >5 cm. Likewise, the prevalence of prolapse increased from 9.7% to 13.4% and 20.2% (Table 4). These results were confirmed in multivariable analyses, where the severity of rectal muscle separation emerged as the strongest determinant of hernia and prolapse (Table 5).

**Table 4:** Association between clinical characteristics, including rectal muscle separation, and hernia/prolapse.

Characteristics	*N*	Hernia		Prolapse	
		*N* (%)	*P*-value	*N* (%)	*P*-value
Rectal muscle separation			**<0** **.** **001**		**<0** **.** **001**
<3 cm	813	257 (31.6%)		79 (9.7%)	
3–5 cm	2,539	1,390 (54.8%)		339 (13.4%)	
>5 cm	1,277	871 (68.2%)		258 (20.2%)	
Age, years			**<0** **.** **001**		**<0** **.** **001**
22–31	392	171 (43.6%)		35 (8.9%)	
32–41	2,558	1,366 (53.4%)		350 (13.7%)	
42–51	1,442	831 (57.6%)		222 (15.4%)	
52–61	219	140 (63.9%)		61 (27.9%)	
≥62	18	10 (55.6%)		8 (44.4%)	
Overweight	1,241	661 (53.3%)		211 (17.0%)	
Mildly obese	401	198 (49.4%)		68 (17.0%)	
Severely obese	116	62 (53.5%)		20 (17.2%)	
Term pregnancies			**<0** **.** **001**		**0** **.** **001**
0	20	9 (45.0%)		2 (10.0%)	
1	1,084	497 (45.9%)		128 (11.8%)	
2	2,693	1,541 (57.2%)		391 (14.5%)	
3	677	388 (57.3%)		122 (18.0%)	
≥4	155	83 (53.6%)		33 (21.3%)	
Kristeller (Yes)	957	518 (54.1%)	0.899	206 (21.5%)	**<0** **.** **001**
Episiotomy (Yes)	1,796	959 (53.4%)	0.304	372 (20.7%)	**<0** **.** **001**
Smoking habits			0.029		0.239
Never-smoker	2,292	1,224 (53.4%)		333 (14.5%)	
Ex-smoker	1,440	824 (57.2%)		197 (13.7%)	
Current smoker	894	468 (52.4%)		145 (16.2%)	
Gestat. diabetes (Yes)	644	340 (52.8%)	0.407	107 (16.6%)	0.127
Hypothyroidism (Yes)	588	326 (55.4%)	0.586	99 (16.8%)	0.101
Steroids in pregnancy (Yes)	339	185 (54.6%)	0.946	46 (13.6%)	0.575

*P*-values were computed by Fisher's exact test or chi-square test for categorical variables and by Mann-Whitney ranksum test for quantitative variables. Significant values are highlighted in bold.

**Table 5:** Multivariable analysis of determinants of hernia and prolapse.

	Hernia		Prolapse	
	OR (95% CI)	*P*-value	OR (95% CI)	*P*-value
Rectal muscle separation				
<3 cm	1 (reference)		1 (reference)	
3–5 cm	**2.80** **(2.32–3.38)**	**<0** **.** **001**	1.31 (0.98–1.76)	0.068
>5 cm	**4.91** **(3.96–6.10)**	**<0** **.** **001**	**2.12** **(1.56–2.89)**	**<0** **.** **001**
BMI				
Underweight	0.91 (0.64–1.30)	0.601	0.67 (0.36–1.23)	0.194
Normoweight	1 (reference)		1 (reference)	
Overweight	**0.79** **(0.67–0.92)**	**0** **.** **002**	**1.26** **(1.02–1.55)**	**0** **.** **032**
Obese	**0.56** **(0.43–0.71)**	**0** **.** **001**	0.87 (0.61–1.24)	0.440
Severely obese	0.76 (0.49–1.18)	0.228	1.20 (0.67–2.14)	0.532
Term pregnancies				
1	1 (reference)		1 (reference)	
2	**1.34** **(1.14–1.58)**	**<0** **.** **001**	1.11 (0.87–1.42)	0.416
3	1.23 (0.98–1.54)	0.074	1.09 (0.79–1.50)	0.559
≥4	1.03 (0.70–1.50)	0.895	1.23 (0.75–2.00)	0.415
Kristeller: Yes vs. No	0.93 (0.78–1.10)	0.395	**1.36** **(1.09–1.70)**	**0** **.** **006**
Episiotomy: Yes vs. No	0.99 (0.85–1.16)	0.912	**2.15** **(1.74–2.66)**	**<0** **.** **001**
Age (per 10-year increase)	**1.24** **(1.12–1.38)**	**<0** **.** **001**	**1.33** **(1.16–1.54)**	**<0** **.** **001**
Current smoker	1.03 (0.86–1.23)	0.779	1.06 (0.83–1.37)	0.626
Gestat. diabetes: Yes vs. No	0.90 (0.74–1.08)	0.252	1.18 (0.91–1.53)	0.203
Hypothyroidism: Yes vs. No	1.03 (0.85–1.25)	0.768	1.16 (0.90–1.52)	0.257
Steroids in pregnancy	1.01 (0.78–1.30)	0.959	1.03 (0.71–1.47)	0.893

Odds ratios (OR) and *p*-values were computed by a logistic model, controlling for all other variables.

Significant values are highlighted in bold.

Nulliparous women were excluded from multivariable analysis.

Other important determinants of hernia were older age and a large number of pregnancies, both in univariable (Table 4) and multivariable analyses (Table 5). Obesity was negatively associated with herniation.

The risk of pelvic organ prolapse increased with advancing age, both in univariable and multivariable analyses. The effect of increasing number of pregnancies on prolapse was less pronounced than the effect on hernia, and it achieved significance only in univariable analysis. Prolapse was less common in underweight women and more prevalent in overweight ones. Both the interventions during delivery, i.e., Kristelller's manoeuvre and episiotomy, significantly increased the risk of prolapse (Table 5).

## Discussion

The main results of the present study are:

(1) DRA severity markedly increases with increasing age, BMI, and number of pregnancies. Moreover, gestational diabetes and Kristeller's manoeuvre during labour are positively associated the severity of DRA, while episiotomy is negatively associated.
(2) Larger separation between rectal muscles is associated with increased risk of pain/discomfort and incontinence for liquid but not solid stools. At variance with Fei et al. (1), the present study found that the severity of DRA is associated with increased risk of urinary incontinence.
(3) Severe DRA is associated with abdominal hernia and, to a lesser extent, pelvic prolapse. Additional risk factors for hernia and prolapse are older age and a large number of pregnancies. Overweight and obesity have opposite effects on the two disorders, as they decrease the risk of hernia and increase the risk of prolapse. Interventions during delivery, i.e., Kristeller's manoeuvre and episiotomy, are associated with a significant increase in the risk of pelvic prolapse but not abdominal hernia.

The role of women's age as a risk factor of DRA is unclear. Age is reported a risk factor by Spitznagle, and most recently by Kaufmann (10, 17). On the contrary, other authors have not confirmed this finding (8, 18). These different results could be explained by ethnic and methodological factors. The study by Sperstad considered a cohort of 300 primiparous women between 19 and 40 years of age, of European ethnicity (8). Wu et al. analyzed a cohort of 644 Chinese women from 18 to 90 in age (18). They used separate cutoff values for DRA in patients under 45 years and over 45 years and, to analyze age as a risk factor, they compared 116 elderly women ≥60 years (18% of the cohort) to young women <45 years (50% of the cohort).

Our results regarding high BMI and number of pregnancies as significant risk factors for DRA severity are consistent with the results of other studies. Both obesity and pregnancy increase abdominal pressure and volume. Moreover, multiple pregnancies, as well as other factors such as lack of exercise, may contribute to cumulative mechanical stress to the connective tissue of the abdominal wall contributing to the development of a DRA (10, 17–20).

Hernia affected 54.4% of the patients with DRA in the present study and the severity of DRA was its main risk factor. Hernias are very common in patients with DRA, and in a recent paper (21) abdominal hernia was over 20 times more frequent in patients affected by DRA compared to general population. Umbilical hernia was the most common type of hernia in women (38%) and presented the highest incidence in their reproductive years (21).

Application of fundal pressure during the second stage of labor (Kristeller's maneuver) is sometimes used to accelerate fetal head delivery. The maneuver is associated with a high risk of pelvic floor injury and consequent onset of prolapse of pelvic organs (22, 23). Given our results, we can assume that the maneuver also induces damage of the linea alba leading to a separation between the rectus abdominis.

Our work supports the view that not only DRA onset but also its severity are mainly affected by conditions leading to an increase in intra-abdominal pressure during gestation and childbirth. In particular, the number of pregnancies, maternal obesity, and the traumatic Kristeller maneuver were among the elements analyzed. These elements may link the severity of DRA with the mechanical effect of increased intra-abdominal pressure.

Pregnancy can lead to DRA through hormonally mediated and/or mechanical effects on the abdominal musculature. During the gestation period, increased levels of relaxin, progesterone, and estrogen soften the connective tissues and weaken the linea alba. This weakening, together with the mechanical strain of the anterior abdominal wall due to the enlarging uterus, can result in a separation of the two rectus abdominis muscle along the linea alba. Moreover, it has been hypothesized that multiple pregnancies may contribute to cumulative mechanical stress to the connective tissue of the abdominal wall contributing to the development of a DRA (3, 17, 24).

Blotta has reported a relationship between collagen types in linea alba specimens, collected both above and below the level of the umbilicus, and DRA. She found that in women with diastasis recti, type I and type III collagen were less abundant than in women without diastasis (25). Others studies found that smoking, which has detrimental effects on fibroblast biology, induces a weakness of the linea alba through a deficit of hydroxyproline, an early compound in the collagen synthesis pathway (26, 27). Despite diabetes can impair wound healing and contribute to develop incisional hernias, its potential role in DRA pathogenesis is not clear (28). In a study by Wu et al. 43.0% of diabetic women had DRA while only 25.5% of non-diabetic women had the abdominal wall disorder (18). In the present study diabetes and gestational diabetes showed a significant correlation with DRA severity, but only in univariable analysis not in multivariable analysis when controlling for BMI and age (data not shown). Among other factors traditionally associated with wall pathology, the present study did not highlight any statistically significant associations between DRA severity and smoking habits, collagen disease, steroid use, and thyroid disease. These findings could therefore frame DRA as a pathology with a predominantly “mechanical” genesis.

Severity of DRA was strongly associated with the frequency of postural disorders such as back pain. It has been reported that DRA adversely affects normal trunk function but the matter is still controversial. In a recent systematic review, a positive correlation between DRA and back pain was observed in only 38.5% of the included studies (29).

Another finding of the present study is that the greater the separation of the recta, the greater the risk of both urinary and fecal incontinence. As described above, physiological urination and defecation functions can be traced back to the correct contractility of the abdominal wall muscles, so their inefficiency is related to the onset of incontinence. Unfortunately, detailed information on this disorder, such as at rest or stress incontinence, are lacking, so little can be said about the underlying abdominal dynamics alteration.

A number of studies reported that the presence of DRA can predispose pelvic floor dysfunctions which include urinary as well as fecal incontinence, although their relationship is still debated (17, 30, 31). Factors such as age, pregnancy, childbirth, type of delivery, number of births, and BMI play a role in the etiology of pelvic floor dysfunctions but are also linked to the onset of DRA (32). As two possible risk factors of DRA are connective tissue weakness and increased intra-abdominal pressure, which are also risk factors for pelvic floor diseases, it can be reasonably assumed that there are common causes. However, a mild effect of DRA severity on urinary incontinence persisted as significant even when controlling for obesity, number of pregnancies, episiotomy. Anyway, further studies are needed to clarify the relationship between DRA and dysfunctions of the pelvic floor.

In recent years some studies have evaluated the effects of surgical treatment of DRA on symptoms such as postural disorders, back pain, and urinary incontinence. The results of these analyses support the role of DRA in determining these symptoms. Indeed, surgical reconstruction of the linea alba can restore the normal anatomy of the abdominal wall with an improvement of abdominal trunk function and urinary incontinence (33–35).

Abdominal hernia and pelvic prolapse were frequent comorbidities in this population of women were rather frequent disorders in the population under study, affecting 54.4% and 14.6% of women with pelvic prolapse. Indeed, DRA severity emerged as the strongest determinant of both abdominal hernia and pelvic prolapse.

### Strengths and limitations

Our study has some limitations. First, the sample was not randomly drawn from the general population but rather originates from a pathology registry, so that it is not possible to estimate prevalence of DRA but only of DRA severity and its determinants. Moreover, participation in the survey was not large, reaching a response rate of about 20%.

In addition, this study did not consider the impact of DRA and associated symptoms on aesthetics and quality of life.

### Conclusions

The prospects arising from our work confirm and possibly consolidate our knowledge on individual risk factors for the development of severe DRA and related disorders. Of course, the associations observed in this cross-sectional study should be confirmed in prospective cohort studies. A subsequent step could be to develop a risk score for predicting the development and/or worsening of DRA, which could allow the implementation of preventive pre-delivery measures.

## Data Availability

The datasets presented in this article are not readily available due to data protection regulation. Data are available from the corresponding author, on reasonable request. Requests to access the datasets should be directed to Giuseppe Verlato, giuseppe.verlato@univr.it.
