# Supplementary material for: Rectus muscle diastasis in Italian women: determinants of disease severity, and associated disorders
Source: Front Surg. 2024 Mar 11;11:1360207. doi: 10.3389/fsurg.2024.1360207 (PMC10961386; doi:10.3389/fsurg.2024.1360207)
Supplement: Supplementary file 1 [file Table1.docx]

**Supplementary Table 1**. Questionnaire used to assess rectus muscle diastasis, administered by Diastasi Donna® ODV (Women’s Diastasis Association).

| Subject id |
| --- |
| Age (Years) |
| Weight (kg) |
| Height (cm) |
| Number of full-term pregnancies: 0-1-2-3- 4 or more |
| How many natural childbirths? (numeric value only) |
| Have you ever had twin pregnancies?: No,Yes  No vs yes) |
| Have you ever undergone the Kristeller’s maneuver?: No, Yes, Don’t know |
| Have you ever had episiotomy/postpartum laceration?: No, Yes, Don’t remember |
| Have you ever had hernias before pregnancy?: No, Yes |
| Do you have collagen disease?: No, Yes, Don’t know |
| Do you have thyroid disease?: None, Hypothyroidism, Hyperthyrodism, Don’t know |
| Smoker: No, Yes |
| Ex-smoker: for <5 years, for 5-10 years, for >10 years |
| Diabetes: No, Yes, Don’t know |
| Gestational diabetes: No, Yes, Don’t know |
| Did you take continuous therapy with corticosteroid before pregnancy?: No, Yes |
| Did you take corticosteroid during pregnancy?: No, Yes |
| Diastasis: Supra-umbilical, sub-umbilical, both |
| pregnancy order at diastasis onset (enter numerical value only) |
| distance of muscles, measured with radiological examination: <3 cm, 3-5 cm, >5 cm |
| presence of hernia: none, umbilical, supra-umbilical, epigastric |
| radiological investigation: ultrasounds, abdominal magnetic resonance imaging, abdominal CT-scan |
| specialist visit: none, general surgeon, plastic surgeon, family physician, physiotherapist |
| Abdominal discomfort/pain |
| intensity: from 0 (no discomfort/pain) to 5 (maximum discomfort/pain) |
| frequency: never, once a day, 2-5 times a day, >5 times a day, all day, always |
| Postural problems/back pain |
| intensity: from 0 (no symptoms) to 5 (maximum symptoms)  0symptom, 5 maximum value) |
| frequency: never, once a day, 2-5 times a day, >5 times a day, all day, always |
| Faecal Incontinence (the question refers only to losses not to normal evacuations) |
| solid stool incontinence: never, seldom, sometimes, often, always |
| liquid stool incontinence: never, seldom, sometimes, often, always |
| gases incontinence: never, seldom, sometimes, often, always |
| using diapers for faecal incontinence: never, seldom, sometimes, often, always |
| Limitations in daily activities for faecal incontinence: never, seldom, sometimes, often, always |
| Urinary incontinence |
| How often do you leak urine?: never, sometimes, usually, always |
| To what extent do urinary leak negatively affect your life overall?: 0-1, 2-3, 4-5, 6-7, 8-9, 10 |
| Under what circumstances do you leak urine?: For no apparent reason, when coughing or sneezing, during sleep, during physical activity, before reaching the toilet, after getting dressed after urination, always |
| Pelvic organ prolapse (certified by a specialist in general surgery / gynecology): No, Yes |
| Have you ever had hysterectomy, adnexectomy?: No, Yes |
| Other symptoms (open question) |
| Symptom remission at 12 months (Without Intervention): No, Yes |
| Symptom remission at 18 months (Without Intervention): No, Yes |
| Diastasis remission at 12-months (without intervention): No, Yes |
| Diastasis remission at 12-months (without intervention): No, Yes |
| Have you ever undergone rehabilitation after pregnancy? No, physiotherapy, postural gymnastics hypopressive gymnastics, |
| I agree to the anonymous publication of my data: No, Yes |
